# Formation of a Neuronal Membrane Model: A Quartz Crystal Microbalance with Dissipation Monitoring Study

**DOI:** 10.3390/biom15030362

**Published:** 2025-03-02

**Authors:** Elaheh Kamaloo, Terri A. Camesano, Ramanathan Nagarajan

**Affiliations:** 1 Department of Chemical Engineering, Worcester Polytechnic Institute, Worcester, MA 01609, USA; elaheh.kamaloo@gmail.com (E.K.); terric@wpi.edu (T.A.C.); 2 US Army Combat Capabilities Development Command Soldier Center, Natick, MA 01760, USA

**Keywords:** supported lipid bilayer, neuronal membrane model, multicomponent lipid vesicle, vesicle rupture, osmotic stress, QCM-D

## Abstract

Supported lipid bilayers (SLBs) that model neuronal membranes are needed to explore the role of membrane lipids in the misfolding and aggregation of amyloid proteins associated with neurodegenerative diseases, including Parkinson’s and Alzheimer’s disease. The neuronal membranes include not only phospholipids, but also significant amounts of cholesterol, sphingomyelin, and gangliosides, which are critical to its biological function. In this study, we explored the conditions for the formation of an SLB, for the five-component lipid mixture composed of zwitterionic 1,2-Dioleoyl-sn-glycero-3-phosphocholine (DOPC), anionic 1,2-dioleoyl- sn-glycero-3-phospho-L-serine (DOPS), nonionic cholesterol (Chol), zwitterionic sphingomyelin (SM), and anionic ganglioside (GM), using the quartz crystal microbalance with dissipation monitoring (QCM-D) technique, by varying experimental parameters such as pH, buffer type, temperature, vesicle size, and osmotic stress. SLB formation from this multicomponent lipid system was found challenging because the vesicles adsorbed intact on the quartz crystal and failed to rupture. For most of the variables tested, other than osmotic stress, we found no or only partial vesicle rupture leading to either a supported layer of vesicles or a partial SLB that included unruptured vesicles. When osmotic stress was applied to the vesicles already adsorbed on the surface, by having a different salt concentration in the rinse buffer that follows vesicle flow compared to that of the dilution buffer during vesicle flow and adsorption, vesicle rupture increased, but it remained incomplete. In contrast, when osmotic stress was applied during vesicle flow and adsorption on the surface, by having different salt concentrations in the dilution buffer in which vesicles flowed compared to the hydration buffer in which vesicles were prepared, complete vesicle rupture and successful formation of a rigid SLB was demonstrated. The robustness of this approach to form SLBs by applying osmotic stress during vesicle adsorption was found to be independent of the number of lipid components, as shown by SLB formation from the 1-, 2-, 3-, 4-, and 5-component lipid systems.

## 1. Introduction

Supported lipid bilayers (SLBs) are widely used models to mimic biological cell membranes because of their simplicity, reproducibility, and being amenable to be characterized by different experimental techniques [1,2,3,4,5,6,7,8]. The most common method of formation of SLBs is vesicle fusion, which involves the transition of spherical vesicles in solution to adsorbed lipid bilayers on a solid surface, through one or more mechanistic steps. Vesicle fusion involves the adsorption of vesicles from the solution on to a solid surface, the fusion and deformation of adsorbed vesicles, followed by vesicle rupture and spreading to form an extended lipid bilayer. Vesicle fusion has been a successful approach to form an SLB when the lipid system is composed of one or two lipid components, as has been extensively demonstrated in the literature [9,10,11,12,13].

The formation of SLBs has been monitored by different techniques such as surface plasmon resonance, ellipsometry, and quartz crystal microbalance with dissipation monitoring (QCM-D). Although these techniques provide information only on the average properties of the SLB formed on a solid surface, they are widely used because there is no non-uniformity in lipid distribution anticipated for the single and binary lipid systems that have been commonly studied, and the average properties represent the local properties as well [8]. Among these techniques, QCM-D monitoring has emerged as the most frequently used approach since the experimental signatures of SLB formation based on QCM-D are very informative and well understood [9,10,11,12,13].

The SLB model composed of single or binary lipid systems captures many essential features of cell membranes and does provide a useful platform for exploring membrane properties [14,15,16,17,18,19,20]. Indeed, in past works in our laboratory, these simple SLB models have been used to differentiate how different antimicrobial peptides destabilize the membrane structure [21,22,23]. Using these SLB models, we have also demonstrated how nanoparticles that may be inherently non-toxic to cells, because of inability to penetrate the cell membrane, can become toxic if other chemicals present in the environment promote nanoparticle entry into the cell membrane [24].

Complex biological membranes are composed of lipids with different polar groups, chain lengths, and saturation states, in addition to incorporating proteins [25,26,27,28]. Further, the lipids may be heterogeneously distributed in the bilayer including microdomains of differing compositions. The composition of the lipids and the resulting microstructure of the membrane control various cellular functions of biological importance. The lipid composition of the brain neuronal cells is believed to enhance or accelerate the susceptibility of the brain to disease states, by playing a significant role in amyloid formation and resultant cellular toxicity [29]. The brain lipids are composed of the following three main categories: glycerophospholipids (major components are zwitterionic phosphatidylcholine (PC), zwitterionic phosphatidylethanolamine (PE), anionic phosphatidylserine (PS), anionic phosphatidylinositol (PI)), sphingolipids (major components are zwitterionic sphingomyelin (SM), the glycosphingolipid, anionic ganglioside (GM1)), and nonionic cholesterol (Chol). In addition to the lipid head group classes mentioned here, the neuronal lipids also have a distribution of lipid chain lengths.

For understanding the fundamental processes of amyloid formation and cellular toxicity through in vitro experiments, we require neuronal membrane models containing all representative lipid components. With such a neuronal lipid bilayer membrane model, one can study various aspects of neuronal function including lipid–protein interactions, neurotransmitter dynamics, membrane fluidity, effects of different lipid compositions on signal transduction, drug interactions with the membrane, the role of lipid rafts in synaptic plasticity, and the impact of disease-associated lipid changes on neuronal function; essentially, investigating how the unique lipid composition of a neuronal membrane affects its behavior and contributes to neurological processes.

While the process of SLB formation from a single lipid or binary lipid mixtures is well established and robust, formation of an SLB from multicomponent lipid mixtures containing typical brain lipids to create a neuronal membrane model has been challenging. Although SLB formation has been reported in a few papers for selected lipid compositions, a robust approach that can be employed for multiple components and compositions is currently lacking. In attempting to form an SLB with a lipid mixture composed of a zwitterionic phospholipid, sphingomyelin, and cholesterol, Sundh et al. [30] observed that the inclusion of sphingomyelin and cholesterol into phospholipid vesicles significantly impaired the vesicle rupture process. When increasing the cholesterol content, the vesicles formed SLBs containing more defects in the form of intact vesicles adsorbed on the surface where vesicles did not break at all but formed supported vesicular layers. For certain compositions of a zwitterionic phospholipid, sphingomyelin, and cholesterol, Melby et al. [31] were able to form SLBs using the vesicle fusion method. However, when we repeated this procedure for the above lipid mixture but after adding an anionic phospholipid to represent brain lipids’ composition, the vesicles simply adsorbed on the surface without rupturing and SLB formation did not occur. Sasahara et al. [32,33] reported the formation of a bilayer for the lipid mixture DOPC/DOPS/SM/Chol with the molar composition (0.4/0.1/0.25/0.25), and including GM1 at different concentrations (0%, 1.3%, 2.5%, 3.8% mol/mol) relative to the total amount of other lipids. We were unable to reproduce the results using the procedure described in their publication.

The goal of this work is to develop a robust approach to SLB formation that can be routinely used for multicomponent lipid systems. The neuronal lipid composition has wide variability [26,28]; therefore, choosing any single lipid mixture composition will never be representative of the many possible compositions that depend on the location of the cells. In all neuronal cells, PC, PE, SM, and cholesterol are key components present at a higher mole percent while PS, PI, and GM1 are important components present at a lower mole percent. Since PC and PE are both zwitterionic glycerophospholipids, we chose to include PC in the lipid mixture, representing both PC and PE lipids. Since PS and PI are both anionic glycerophospholipids, we chose PS to represent both PS and PI lipids. The other major components included are the sphingolipid, zwitterionic SM, the glycosphingolipid, anionic GM1, and nonionic cholesterol (Chol). As for the relative amounts of lipid components, we selected the lipid mixture composition that has been used for the neuronal membrane model in earlier works [32,33], since that is the only 5-component lipid mixture that had been tried on the QCM-D platform. Future experiments could explore broader compositional variations such as larger cholesterol concentrations or including PE along with PC.

Since SLB formation by vesicle fusion involves vesicle adsorption on the substrate and vesicle rupture, we explored how to facilitate each of these processes to promote the formation of an SLB from this complex lipid mixture. Vesicle adsorption is influenced by vesicle–substrate and vesicle–vesicle interactions which can be manipulated by changing solution properties such as solvent composition, pH, ionic strength, etc. Vesicle rupture has been proven to be a response to release the high curvature energy of vesicles. Parameters such as vesicle size, osmotic stress, and ionic concentration can influence the rupture since they directly affect the bending energy of the vesicles and the vesicle tension at the substrate–vesicle contact area. Each of these experimental variables has been successfully exploited for forming SLBs from vesicles by vesicle fusion. Here, we explore these experimental variables as to their effectiveness in promoting SLB formation from the complex multicomponent lipid mixture to create a neuronal membrane model.

## 2. Background to SLB Formation and Monitoring by QCM-D

### 2.1. Mechanistic Steps in SLB Formation

**Figure 1:**
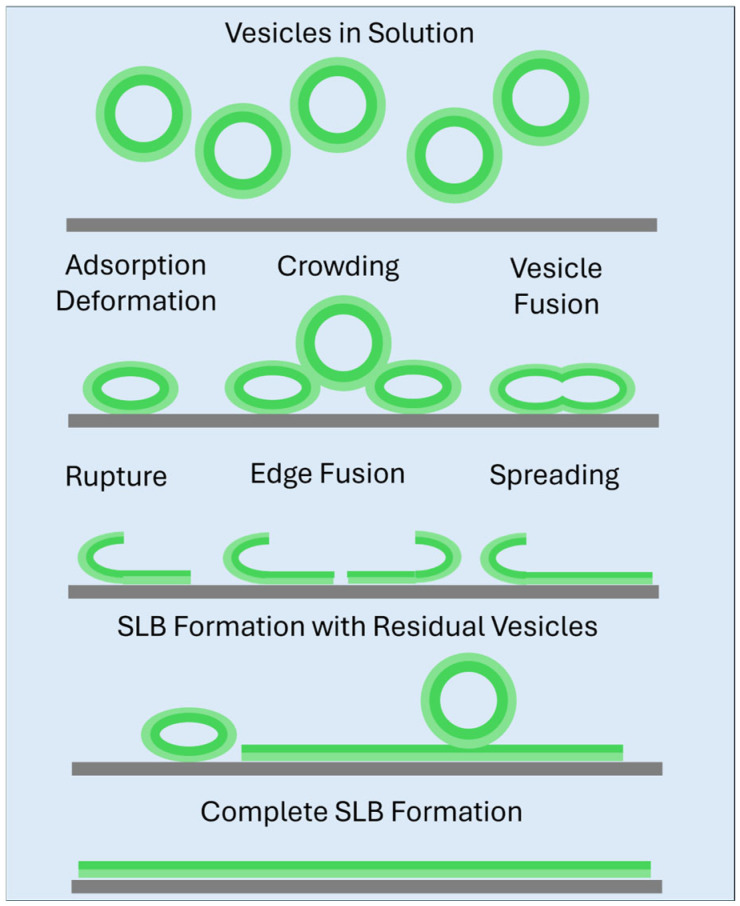
Mechanism of supported lipid bilayer (SLB) formation on quartz surface from vesicles suspended in aqueous medium. The inner and outer layers of the vesicles are shown in different shades of green. The various mechanistic steps—vesicle adsorption, deformation, vesicle fusion, rupture, edge fusion, spreading—are discussed in the text. Adapted from Anderson et al. [35].

Various processes that may occur leading to the formation of a supported lipid bilayer when a solution of vesicles comes into contact with a surface have been discussed in the literature [34,35,36,37]. A schematic representation presented by the Israelachvili lab [35] is shown in Figure 1. Under sufficiently adhesive conditions, isolated vesicles from the solution can adsorb to the substrate surface. If the adhesion is strong enough or the vesicle is in a stressed state (e.g., osmotically), the isolated vesicle can deform and generate intra-bilayer stresses sufficient to promote vesicle rupture, forming a bilayer island on the surface [34]. If the strength of adhesion is not strong enough to rupture isolated vesicles on the surface (as is the case with PC bilayers on silica), the vesicles require additional stresses from neighboring vesicles to cause rupture, that is, a critical vesicle concentration on the solid substrate. After the initial rupture of vesicles, subsequent vesicles can fuse with the unfavorable edges of the bilayer patches, through hydrophobic interactions between the tails of the lipids in the highly curved regions of the bilayer edge and the stressed vesicle [36]. This process continues until the bilayer is complete, at which point excess lipid and water may be ejected back into solution.

Anderson et al. [35] determined that the electrostatic double-layer interaction is the most important factor leading to the strong attraction and adhesion of zwitterionic bilayers to anionic hydrophilic surfaces such as silica or quartz crystal. The van der Waals attractive force was estimated to be relatively weak and inadequate to induce sufficient stresses to cause rupture, which is a prerequisite for the lipid spreading on the surface. Indeed, the importance of electrostatic interactions was confirmed by the inability of anionic lipid vesicles to adsorb and form an SLB on the negatively charged quartz surface [38]. However, the anionic lipid vesicles are shown to readily adsorb and form SLBs on a quartz surface functionalized with trimethoxy aminosilane groups to make the surface cationic, further indicating the importance of the electrostatic interactions. In contrast to the behavior of fully anionic lipid vesicles, if the vesicle is composed of a mixture of anionic and zwitterionic lipids, studies in the literature have demonstrated spontaneous SLB formation by vesicle fusion for up to 20 mole percent concentration of the anionic lipid in the mixture [38]. This indicates that the anionic lipid–anionic substrate electrostatic repulsions are modulated by the presence of the zwitterionic lipids in the system, up to a limited compositional range of anionic lipids.

### 2.2. QCM-D Monitoring of SLB Formation

QCM-D has been widely used as a powerful analytical tool for the real time investigation of the details of vesicle-to-bilayer transition [2,3,10,11,12,13]. In the QCM-D experiments, changes in frequency (ΔF) and energy dissipation (ΔD) of an oscillating sensor crystal are measured (relative to a base line without lipids) as mass attaches to and/or is removed from the surface of the crystal, during the vesicle adsorption, rupture, and SLB formation process. A decrease in frequency can be related to an increase in mass on the crystal surface and vice versa and the crystal oscillating with a natural frequency of 5MHz has a mass sensitivity of ∼1.8 ng/cm^2^. Changes in dissipation can be related to changes in the viscoelasticity, or “softness”, of the membrane on the sensor. Since the disordering of lipids within a membrane can introduce spaces that would weaken the structure, dissipation measurements can reveal information about the level of disruption in a bilayer on the sensor surface.

QCM-D measures frequency and dissipation changes not only at the fundamental resonant frequency of the quartz crystal, but also in its higher harmonics. Due to different penetration depths of the acoustic wave associated with different overtones, higher overtones (e.g., the 11th harmonics) are associated with activity near the crystal surface and lower overtones (e.g., the 3rd harmonics) are related to processes occurring near the external surface of the attached mass, in contact with the bulk liquid. It should be noted that the ΔF values are automatically normalized to each overtone so that ΔF is the change in F_n_/n, where n is the harmonic number and F_n_ is the frequency at the nth harmonic. When a rigid SLB forms, the frequency and dissipation changes at all overtones converge to effectively a single value of ∆F and ∆D. In contrast, when a viscoelastic film of partial bilayer accommodating some intact vesicles forms, the ∆F and ∆D values at various overtones diverge.

The relation between the experimentally observable variables of frequency change ∆F and dissipation change ∆D to the mass of the film and its viscoelastic properties has been well established in the literature [39,40]. For a rigid film on the crystal surface when the crystal is in air, the frequency change ∆F and the areal mass of the film deposited on the crystal, m_f_ (mass per unit area) are related by the Sauerbrey equation. The dissipation change ∆D, by definition, is zero.

(1) $$∆F={-F}_{o}\frac{m_{f}}{m_{q}},\;∆D=0,\;m_{f}=∆m=-C∆F$$

Here, F_o_ is the natural frequency of the oscillator, m_q_ is the areal mass of the quartz crystal, and C is a proportionality constant equal to 17.8 ng/cm^2^/Hz for a crystal with a natural frequency of 5 MHz. The mass addition (∆m) due to the film deposited on the crystal surface gives rise to a decrease in the frequency (negative ∆F). Note that the use of the Sauerbrey equation is strictly valid for a rigid film on the quartz crystal.

For a rigid film on the crystal surface when the crystal is immersed in a Newtonian liquid (like water), the frequency and dissipation changes are modified due to the presence of water and are given by the following:

(2) $$∆F=-\frac{\eta_{L}}{2\pi \delta_{L}m_{q}}-F_{o}\frac{m_{f}}{m_{q}},\;∆D=\frac{\eta_{L}}{n\pi F_{o}\delta_{L}m_{q}}$$

where η_L_ is the viscosity of the liquid medium and δ_L_ is the decay length of the acoustic wave in the liquid medium. The first term in the expression for ΔF and the only term appearing in ΔD are due to the solvent effect because of the immersion of the crystal in the liquid and they vanish when we consider the changes in the crystal properties before and after the deposition of the rigid film on the crystal surface (since we are measuring the difference between two states). Effectively, the film mass changes are given just by the Sauerbrey term and ΔD is zero.

**Figure 2:**
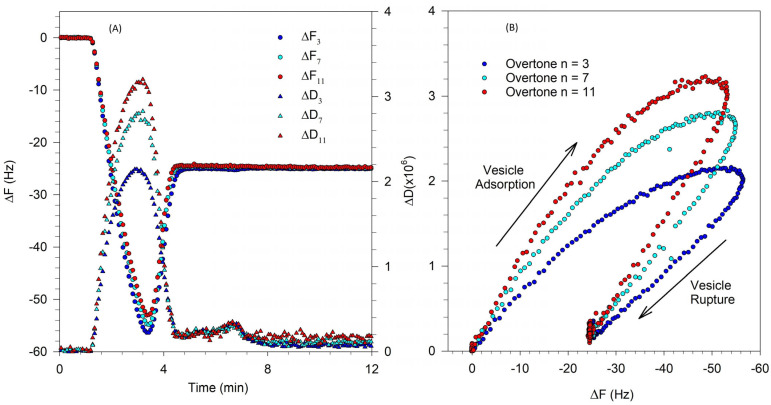
SLB formation from DOPC vesicles by vesicle fusion process. (**A**) Change in frequency ΔF and dissipation ΔD corresponding to the 3rd, 7th, and 11th overtones of the natural frequency of the quartz crystal. The asymptotic frequency change of about −25 Hz corresponds to the formation of a rigid SLB. The film is rigid as confirmed by the very small asymptotic value for the change in dissipation. (**B**) The time evolution plot of ΔD vs. ΔF allows one to infer qualitatively the nature of dynamics controlling the SLB formation. The points shown in this plot represent ΔF and ΔD values at evenly spaced time intervals. Therefore, larger spacing between points indicate that the mass or viscoelasticity changes in the film occur at a faster rate. Changes in slope in this plot generally indicate a change in mechanism, with vesicle adsorption occurring initially during which the magnitudes of both ΔF and ΔD increase, followed by vesicle rupture, during which the magnitudes of both ΔF and ΔD decrease.

If the film is not rigid but viscoelastic, then the frequency and dissipation changes are both influenced by the viscoelastic properties. The dissipation D is the ratio between the energy loss per oscillation period and the total energy stored and is related to the loss modulus G″ and the storage modulus G′ in the form, D = G″/(2πG′). The change in dissipation ∆D is thus directly related to the viscoelasticity of the film attached to the crystal surface. The change in frequency and dissipation for a viscoelastic film are given by the following:

(3) $$∆F=-\frac{\eta_{L}}{{2\pi \delta }_{L}m_{q}}{-F}_{o}\frac{m_{f}}{m_{q}}\left[1-\frac{2}{\rho_{f}}{\left(\frac{\eta_{L}}{\delta_{L}}\right)}^{2}\frac{G\prime \prime }{{G\prime }^{2}+{G\prime \prime }^{2}}\right],\;∆D=\frac{\eta_{L}}{n\pi F_{o}\delta_{L}m_{q}}+\frac{m_{f}}{m_{q}}\left[\frac{4}{\rho_{f}}{\left(\frac{\eta_{L}}{\delta_{L}}\right)}^{2}\frac{G\prime }{{G\prime }^{2}+{G\prime \prime }^{2}}\right]$$

where ρ_f_ is the density of the film on the crystal surface. As in Equation (2), the first term in the expressions for ΔF and ΔD are due to the solvent effect and they vanish when we consider changes in film properties when the crystal is immersed in the liquid both before and after exposure to the vesicle solution. The second term in ΔF is the Sauerbrey contribution that is already present in Equation (2) and the third term in ΔF accounts for a correction to the Sauerbrey mass coming from the viscoelasticity of the film. The second term in ΔD is the direct contribution to dissipation arising from the viscoelasticity of the film. An increase in ∆D corresponds to a decrease in the storage modulus G′ and indicates a less rigid, possibly more disordered film. A decrease in ∆D corresponds to an increase in the storage modulus and indicates a more rigid film on the crystal surface. As mentioned earlier, when a rigid SLB forms, the frequency and dissipation changes at all overtones converge to effectively a single value of ∆F and ∆D, while when a viscoelastic film of partial bilayer accommodating some intact vesicles forms, the ∆F and ∆D values at various overtones diverge. The characteristic QCM-D signatures associated with the formation of SLB from vesicles are shown in Figure 2, for experiments conducted with a single component zwitterionic lipid, DOPC.

Figure 2A shows the change in frequency ΔF and dissipation ΔD corresponding to the 3rd, 7th, and 11th overtones of the natural frequency of the quartz crystal. Lipid vesicles present in solution first attach to the QCM-D sensor surface. The attachment of water-filled vesicles with water trapped between them in the film is indicated by the sharp lowering in the frequency and the large increase in the dissipation. Note the divergence of various overtones at this frequency minimum and dissipation maximum. Once a critical surface concentration of adsorbed vesicles is reached, as indicated by the minimum in the frequency change, the vesicle rupture, releasing the fluid within them and creating a supported lipid bilayer. As the bilayer forms, dissipation decreases because the bilayer is more rigid than the film of water-filled vesicles. The frequency and dissipation changes at various overtones converge and a complete rigid SLB is characterized by a ΔF of ∼25 Hz and ΔD less than 0.5 × 10^−6^.

Figure 2B shows the relation between ΔD vs. ΔF and provides a qualitative inference on the dynamics of the vesicle-to-SLB transition. Each point represents the values for ΔD and ΔF at a given time instant. Traces pointing east reveal increases in mass while those pointing north reveal an increase in softness or viscoelasticity. The points shown in this plot represent ΔF and ΔD values at evenly spaced time intervals. Larger separation between points indicates that the mass or viscoelasticity changes in the surface film occur at a faster rate. Changes in slope in this plot generally indicate a change in mechanism. As the vesicles attached to the QCM-D sensor surface, the mass on the sensor increased, causing the frequency to sharply decrease. This process is shown in the ΔD vs. ΔF plot as the points move in the northeast direction (shown by the arrow labeled vesicle adsorption), indicating an increase in mass on the surface as well as the viscoelastic nature of adsorbed vesicle layer. As the vesicles rupture and organize into a planar bilayer, there is a mass loss due to the release of water from the vesicle interior as well as the loss of excess lipid from the vesicle. Simultaneously, there is a decrease in dissipation change due to the higher ordering of the planar bilayer compared to the soft water-filled vesicles. Correspondingly, the ΔD vs. ΔF trace changes in direction (labeled vesicle rupture), resulting in a southwest trend that indicates a loss in mass and a decrease in viscoelasticity. The data from the 3rd, 7th, and 11th overtones show similar behavior and extend over the same range of ΔD and ΔF, both being large. This suggests that the bilayer formation process starting from adsorbed vesicles causes identical changes both near the crystal surface (11th overtone) as well as at the interface with bulk water (3rd overtone).

### 2.3. Use of QCM-D Data to Determine Vesicle Deformation

**Figure 3:**
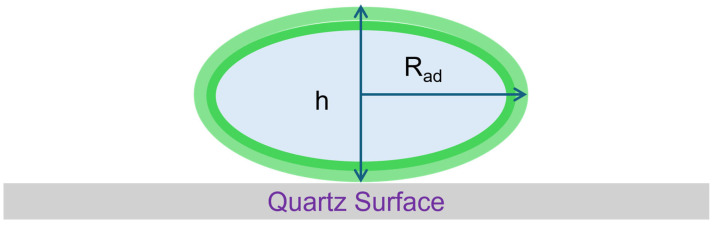
Deformation of vesicle adsorbed on the quartz surface. The height h of the deformed vesicle above the surface will be smaller than that of the undeformed vesicle and a method to determine h from QCM-D data is described in the text.

It has been previously proposed in the literature that vesicles adsorbed on the surface possess a pancake-like shape [41,42,43,44], which is schematically shown in Figure 3. The shape of the adsorbed vesicle is determined by the competitive factors of the adsorption energy of the vesicle with the quartz substrate, curvature energy associated with the bilayer bending, membrane tension due to the areal changes in the bilayer, and osmotic stress due to the volume changes in the vesicle.

**Figure 4:**
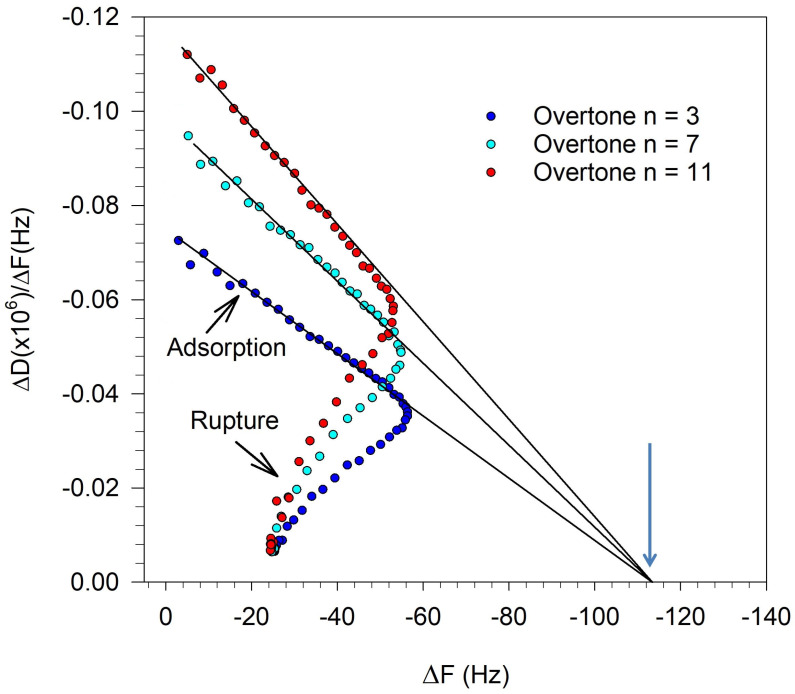
Determining the height of the deformed vesicle by the approach proposed by Reviakine et al. [43]. ΔD and ΔF values used in this plot at various overtones correspond to those shown in Figure 2 for SLB formation from DOPC vesicles. The extrapolation of the data to obtain the intercept used the portion of the QCM-D trace representing vesicles adsorbing on the quartz crystal surface. Arrow indicates the ΔF value at the intercept.

An approach to determining the height of the deformed vesicles on the surface from the QCM-D data has been proposed and validated by Reviakine et al. [43]. Since QCM-D measures the average properties on the quartz crystal surface, the layer of adsorbed vesicles on the surface is treated as a uniform film of thickness h. If the Sauerbrey relationship is applicable, then the thickness h of the film could be directly determined from the frequency shift ΔF using the relation

(4) $$h=\frac{m_{f}}{\rho }-\frac{C∆F}{\rho }$$

where m_f_ is the mass of the adsorbed film; C is the Sauerbrey coefficient (C = 17.8 ng/cm^2^/Hz); and ρ is the film density (ρ ≈ 1 g/cm^3^). However, as discussed earlier, for the Sauerbrey relation to be valid, the dissipation ΔD should be zero at all overtones and the frequency change ΔF should be equal at all overtones. Neither of these conditions are valid when the vesicles are adsorbed on the crystal surface, corresponding to the minimum in the frequency change, as shown in Figure 2. To obtain the thickness of the film, Reviakine et al. [43] have suggested an approach based on plotting the ratio ΔD/ΔF as a function of the frequency change ΔF, for all the overtones. Such a plot is shown in Figure 4 for the QCM-D data presented in Figure 2. Measurements performed at various overtones extrapolate to a common intercept on the frequency change axis where the dissipation change is zero. We thus satisfy the condition that for all overtones the dissipation is zero and the frequency change values are identical. This allows the use of the Sauerbrey equation to determine the film thickness h, taking this frequency change value at the intercept. In this example, we determine the frequency change at the intercept to be −113 Hz and correspondingly, from Equation (4), the film thickness h is estimated to be 20.1 nm. This approach is used in this study to determine the thickness of the adsorbed vesicles layer, which provides a measure of the size of the deformed vesicle.

## 3. Experimental Procedure

### 3.1. Vesicle Preparation

**Figure 5:**
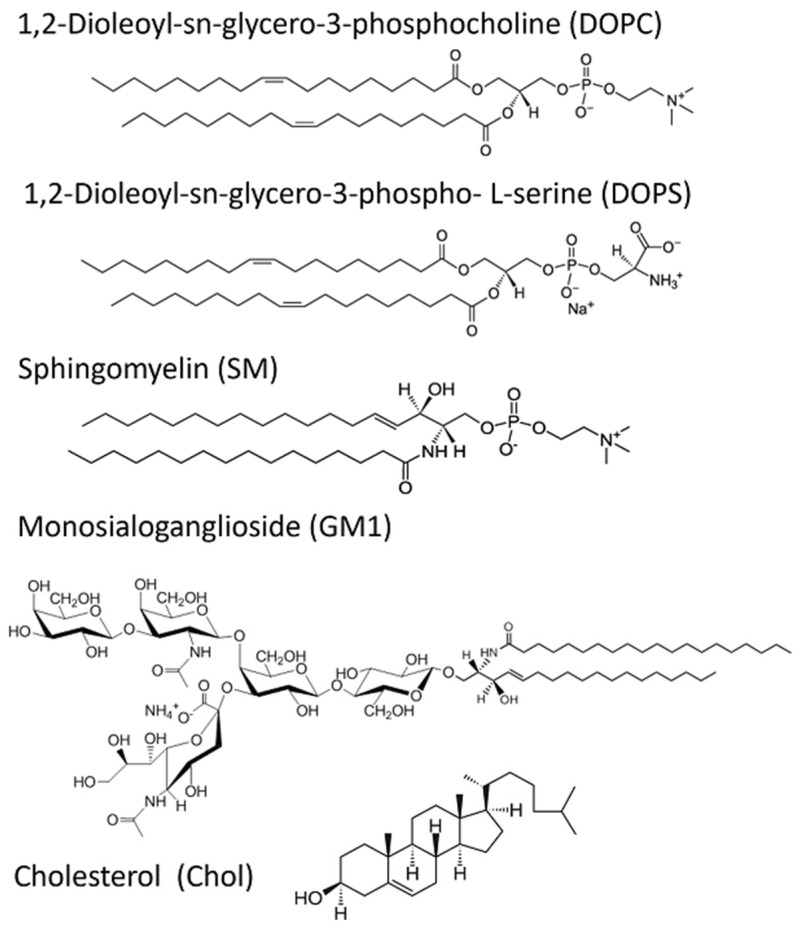
Chemical structures of lipid molecules that are representative components of neuronal membranes and considered in this study. The actual lipid composition varies for specific cells and cell regions within the brain and the lipids depicted here are present in all cases, although at varying compositions. Other lipids not shown here but are present in some appreciable concentrations include zwitterionic phosphatidylethanolamine (PE) and anionic phosphatidylinositol (PI). While the lipid head group classes are represented in this figure, the neuronal lipids also have a distribution of lipid chain lengths [26,28].

For reasons discussed earlier, we chose to reproduce the experimental conditions used by Sasahara et al. [32,33] in terms of the type of lipids and the composition of the lipid mixture. 1,2-Dioleoyl-sn-glycero-3-phosphocholine (DOPC), 1,2-dioleoyl-sn-glycero-3-phospho-L-serine (DOPS), sphingomyelin (SM; egg, chicken), cholesterol (Chol; ovine wool), and monosialoganglioside (GM1; brain, ovine-ammonium salt) were purchased from Avanti Polar Lipids, Inc. (Alabaster, AL, USA). According to the vendor, the transition temperatures of DOPS and DOPC were −11 °C and −17 °C, respectively. Egg sphingomyelin was mostly 16:0 sphingomyelin with a small percentage of 18:0 and 22:0 sphingomyelin. The transition temperatures for SM 16:00, 18:00, and 22:00 were reported by the vendor to be 40.5 °C, 45 °C, and below 47.5 °C, respectively. The chemical structures of the lipid components used in this study are shown in Figure 5.

DOPC, DOPS, SM, and Chol were dissolved in chloroform to a concentration of 10 mM, and GM1 was dissolved in methanol to a concentration of 0.63 mM. The lipids were mixed with the molar ratio of DOPC/DOPS/SM/Chol/GM1; 0.4/0.1/0.25/0.25/0.03. This molar ratio was used for all experiments involving the 5-component lipid mixture in this study. For experiments involving 2-, 3-, or 4-component lipid mixtures, also conducted in this study, the same relative proportion of lipids as in the 5-component lipid mixture were used. After evaporation of organic solvents under a stream of nitrogen, the dried lipid film was kept in vacuum desiccator for at least 4 h to remove any residual solvents. The film was hydrated in the buffer solution (referred to as hydration buffer in the text) at 56 °C to yield the stock solution of vesicles with a total lipid concentration of 0.3 mM.

After five freeze–thaw cycles of the hydrated lipid film, large multilamellar vesicles were formed. To reduce the size of vesicles, extrusion or sonication or a combination of the two techniques were applied. The rotational and translational diffusion studies of vesicles in the literature confirm that there are no differences between the vesicles prepared by sonication and the ones prepared by extrusion [45]. For extrusion, the lipid suspension was extruded 21 times through a polycarbonate filter (pore size, 50 nm) using a mini extruder (Avanti Polar Lipids, Inc.) just before use. For sonication, an ultrasonic dismembrator (Model 150T, Thermo Fisher Scientific, Waltham, MA, USA) was used in pulsed mode for 45 min at 56 °C. A 30% duty cycle was used for sonication (pulse on for 3 s, followed by a pause for 7 s) at an amplitude of 60%. After sonication, the vesicle solution was centrifuged at 16,000 relative centrifugal force (RCF, multiples of gravitational force g) for 10 min to remove any TiO_2_ particles from the ultrasonic dismembrator probe (J2-MI Centrifuge, Beckman Coulter, Brea, CA, USA) [45]. The supernatant containing small unilamellar vesicles (SUVs) was collected and stored at 4 °C under nitrogen and they were stable for two to three weeks. Dynamic light scattering (DLS) was used to determine the average size of the vesicles (Zetasizer Nano ZS, Malvern, Worcestershire, UK). The stock solution of vesicles was diluted using a buffer (referred to as the dilution buffer in the text) to the desired lipid concentration before each QCM-D experiment.

Three different buffer designations are used in this text, as follows: hydration buffer is the one in which the vesicles are prepared, dilution buffer is the one in which vesicle flow and adsorption takes place in the QCM-D, and rinse buffer is that which follows the end of the vesicle flow. Six different buffers designated as B1, B2, B3, B4, B5, and B6 were used either as hydration buffers, dilution buffers, or as rinse buffers. Basically, they were either phosphate buffers or Tris buffers, differing from one another in their salt (NaCl, CaCl_2_, or MgCl_2_) concentrations. The buffer compositions included (B1) 20 mM phosphate + 100 mM NaCl, pH = 7.8; (B2) 20 mM phosphate + 100 mM NaCl, pH = 7.0; (B3) 10 mM Tris + 150 mM NaCl + 2 mM CaCl_2_, pH = 7.40; (B4) 20 mM phosphate + 100 mM NaCl + 2.5 mM MgCl_2_, pH = 7; (B5) 20 mM phosphate + 250 mM NaCl + 2.5 mM MgCl_2_, pH = 7; (B6) 20 mM phosphate, pH = 7. The phosphate buffers were prepared using appropriate amounts of monobasic potassium or sodium dihydrogen phosphate and dibasic potassium or sodium monohydrogen phosphate. Tris buffer solutions were prepared by dissolving appropriate amounts of Tris(hydroxymethyl) amino methane, (Tris base) in water and adjusting the pH with hydrochloric acid.

### 3.2. QCM-D Experiments of SLB Formation

QCM-D data were obtained by a Q-Sense E4 series (Q-Sense AB, Gothenburg, Sweden) instrument with silicon dioxide-coated crystals (5 Hz) purchased from Biolin Scientific (Gothenburg, Sweden). In the cleaning step, the crystals were rinsed with sequential flows of ethanol, deionized water, and 2% sodium dodecyl sulfate and de-ionized water each for 5 min. After that, crystals were thoroughly dried with nitrogen gas and etched with two cycles of 45 s oxygen plasma cleaning using Plasma Prep II (SPI Supplies, West Chester, PA, USA). The experimental procedure began with setting a baseline in the dilution buffer. To form a stable SLB on a crystal, the lipid solution is injected into the QCM-D chamber at 0.15 mL/min to allow vesicles to attach to the silica-coated crystal surface. After the vesicles ruptured to form a stable bilayer, the crystal was rinsed with buffer (referred to as rinse buffer in the text) to remove unattached lipid vesicles and excess molecules [3,46,47,48].

All the experimental systems reported in this paper are summarized in Table 1. The system code refers to the figure number where the corresponding QCM-D results are plotted. The 5-component lipid mixture DOPC/DOPS/SM/Chol/GM1 in all experiments was at the mole ratio 0.40:0.10:0.25:0.25:0.03. For experiments involving 2-, 3-, or 4-component lipid mixtures, the same relative proportion of lipids as in the 5-component lipid mixture was used. Namely, for DOPC/DOPS, the mole ratio was 0.40:0.10, for DOPC/DOPS/SM, the mole ratio was 0.40:0.10:0.25, for DOPC/DOPS/GM1, the mole ratio was 0.40:0.10:0.03, and for DOPC/DOPS/SM/Chol, the mole ratio was 0.40:0.10:0.25:0.25. The hydration buffer used for preparing the vesicle stock solution, the dilution buffer used for the lipid flow, and the rinse buffer used for the following rinse step are all specified.

For this study designed to examine the effect of osmotic stress as the variable, two types of experiments were conducted. In the first case, the osmotic stress was applied after the vesicles were adsorbed on the surface. The vesicles were first made in the hydration buffer to a total lipid concentration of 0.3 mM (correspondingly, the osmolality of the vesicle interior is defined by that of the hydration buffer) and then diluted with the dilution buffer to obtain a final lipid concentration of 0.1 mM (correspondingly, the osmolality of the vesicle exterior is defined by that of the dilution buffer). Both the hydration and dilution buffers in this type of experiment had the same salt concentration and therefore the vesicle adsorption occurred under isosmotic condition. After the end of lipid flow, the vesicles adsorbed on the quartz crystal were rinsed with the rinse buffer that had a different salt concentration, and the osmotic stress was introduced at this stage. In the second type of experiment, the osmotic stress was applied during the adsorption of vesicles on the crystal surface. To enable this, the vesicles made in the hydration buffer were diluted with the dilution buffer that had a different salt concentration compared to the hydration buffer. The osmotic stress was thus introduced during lipid flow, as the vesicles were adsorbing on the crystal surface. The vesicles were diluted in the dilution buffer just before the QCM-D lipid flow to ensure that the osmotic stress exposure time remains consistent for all experiments. The rinse buffer was at the same salt concentration as the dilution buffer. All the experiments were performed in at least triplicate, unless specified otherwise.

## 4. Results and Discussion

### 4.1. 5-Component Lipid Vesicles Remained Unruptured on the Quartz Crystal Surface

**Figure 6:**
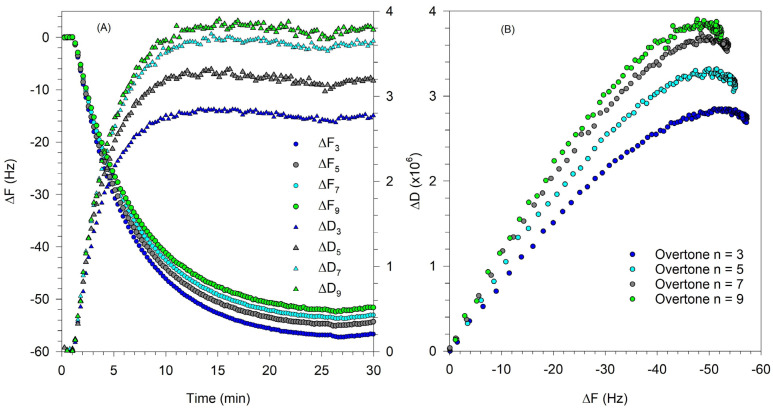
Lipid mixture representing neuronal lipids forming supported lipid vesicles (SLVs) layer. Experimental conditions are listed in Table 1. Vesicles of size ~80 nm and a phosphate buffer system were used. (**A**) QCM-D frequency and dissipation changes as a function of time for the lipid system composed of DOPC/DOPS/SM/Chol/GM1 (0.4:0.1:0.25:0.25:0.03) at 23 °C. The circles represent frequency changes, and the triangles represent dissipation. The plots are shown for various overtones. The asymptotic large magnitudes of the frequency and dissipation changes and the divergence of the overtones indicate that a supported lipid vesicle (SLV) layer is formed and not an SLB. (**B**) The ΔD-ΔF plot shows only the single process of vesicle adsorption taking place and absence of the vesicle rupture process.

The 5-component, DOPC/DOPS/SM/Chol/GM1 (mole ratio 0.40:0.10:0.25:0.25:0.03) lipid vesicles of about 80 nm in diameter were prepared in buffer B1 (20 mM phosphate + 100 mM NaCl, pH = 7.8). The vesicles were diluted in the same buffer B1 and were allowed to flow over the quartz crystal. The vesicle flow was followed by flow of the rinse buffer B1, having the same composition as the hydration and dilution buffers. The QCM-D results are shown in Figure 6.

The QCM-D data in Figure 6A show that vesicles adsorb on the quartz crystal, causing an increase in the magnitude of the frequency change. Following adsorption, the vesicles remained intact without rupture, as shown by the absence of any minimum in the frequency change and the absence of a subsequent decrease in the magnitude of the frequency change. The large values for the asymptotic ΔF and ΔD and their spread over different overtones imply that the surface film consists of soft, water-containing vesicles adsorbed on the quartz surface, with water trapped between the vesicles. The experiments thus indicate the formation of a supported layer of vesicles (SLVs) instead of a supported lipid bilayer (SLB). The ΔD-ΔF plot in Figure 6B when compared to Figure 2B makes clear that the process had only one mechanistic step, namely that of vesicle adsorption.

### 4.2. Change in Buffer from Phosphate to Tris Led to Some Small Amount of Vesicle Rupture

As mentioned earlier, Melby et al. [31] were able to form an SLB using the vesicle fusion method for a lipid mixture of DOPC/SM/Chol. In their experiments, a Tris buffer was used and therefore we conducted QCM-D measurements using a Tris buffer, in place of the phosphate buffer, for the 5-component lipid mixture, DOPC/DOPS/SM/Chol/GM1 (0.40:0.10:0.25:0.25:0.03). The Tris buffer is most effective at a slightly alkaline pH range (around 7–9) due to its pKa value, while a phosphate buffer is generally better for maintaining a near-neutral pH (around 7). Obviously, the chemical composition of the buffering molecule itself is different for the two buffers. It was of interest to see whether such a change in the chemical composition of the buffer while maintaining the same pH will make a difference. Further, experiments were performed with two different sizes of vesicles (81 nm and 108 nm diameters). Since the vesicle size influences the bending energy of the vesicles and thereby the level of deformation after vesicle adsorption, observing the behavior of vesicles of two different sizes was of interest.

**Figure 7:**
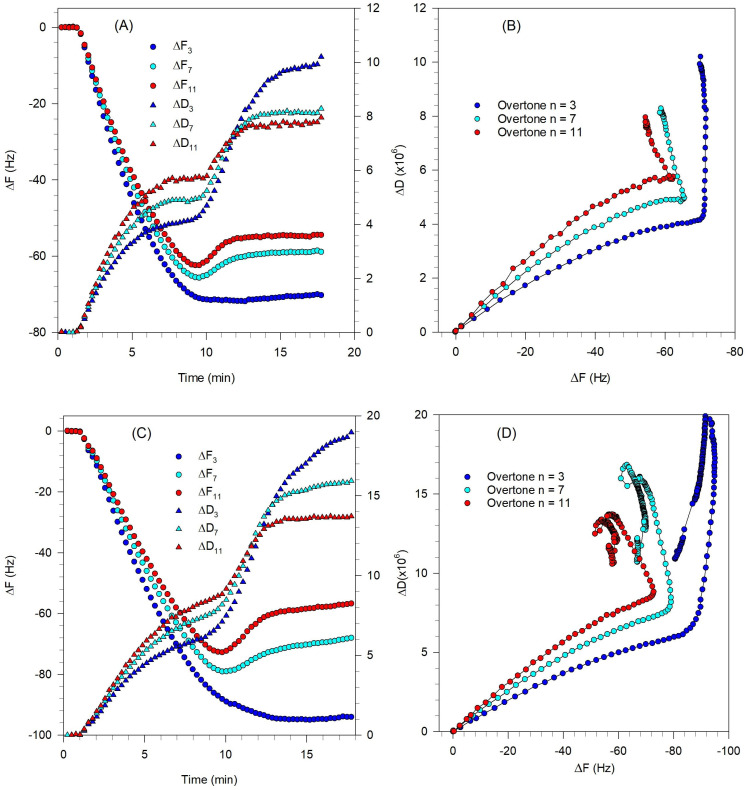
Effect of Tris buffer and vesicle size on the formation of bilayer composed of DOPC/DOPS/SM/Chol/GM1 (0.4:0.1:0.25:0.25:0.03) at 23 °C. Experimental conditions are listed in Table 1 and a Tris buffer system was used. (**A**,**B**) are for vesicles of diameter 81 nm, while (**C**,**D**) are for vesicles of diameter of 108 nm. (**A**,**C**) are the QCM-D frequency and dissipation changes as a function of time, for the different overtones. The circles represent frequency changes, and the triangles represent dissipation. (**B**,**D**) are the ΔD-ΔF plots showing the process of vesicle adsorption taking place and negligible fraction of vesicles rupturing.

The QCM-D results shown in Figure 7A,C indicate that for both the vesicle sizes, only minimal vesicle rupture occurred. While a minimum in the frequency change is observed indicating some vesicle rupture, the extent of the change is very small and the frequency change at the asymptotic condition is not too different from the frequency change at the minimum, indicating only a few vesicles have ruptured. The QCM-D measurements show that the asymptotic values of ΔF and ΔD are much larger than those for a complete SLB and they are spread out for different overtones indicating that the final adsorbed film is soft and dissipative. For both vesicle sizes, the adsorption duration was about 10 min; however, the absolute values of ΔF and ΔD at the critical coverage (minimum in the frequency change plot) are lower for the 81 nm diameter vesicles compared to the 108 nm diameter vesicles. The change in ΔF and ΔD in the adsorption process is more significant for larger vesicles indicating adsorption of more mass on the crystal.

The ΔD-ΔF plots in Figure 7B,D show that the adsorption kinetics for vesicles of both sizes are qualitatively similar indicating the occurrence of a two-step process. The first process, in which the adsorption of intact vesicles is the dominant process, is similar to the first step in the bilayer formation fingerprint (Figure 2B). However, the second step is different from the bilayer formation fingerprint. In Figure 2B, the second step represents the vesicle rupture and is indicated by a process tending in the south–east direction. In contrast, in Figure 7B,D, the second process tends towards north or north–east corresponding to an increase in the softness of the layer. This is indicative of a partial rupture of the vesicles which leads to the hydration and restructuration of a viscoelastic lipid film.

### 4.3. Increase in Temperature Promotes Partial Vesicle Rupture

**Figure 8:**
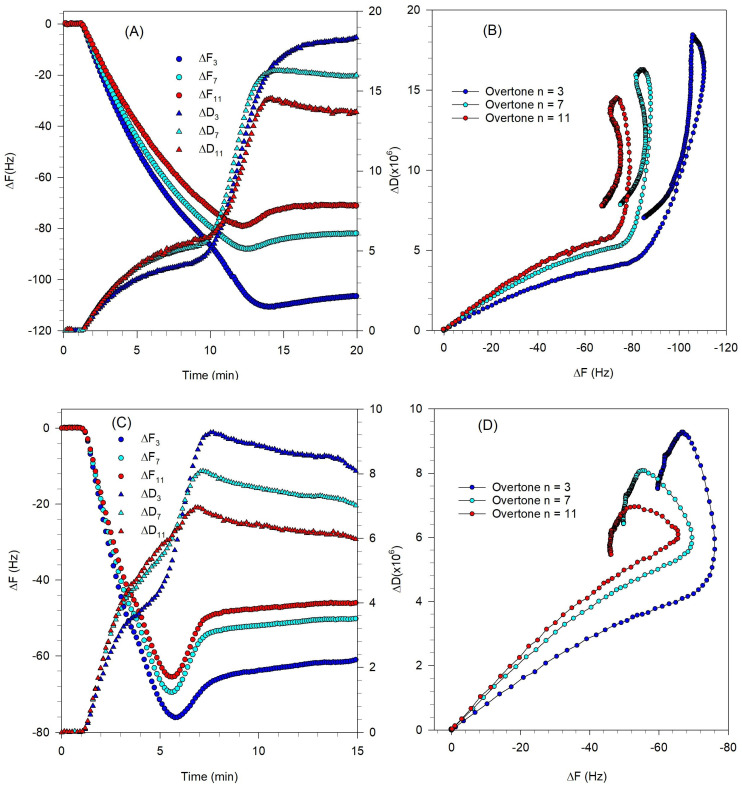
Effect of temperature on the formation of bilayer composed of DOPC/DOPS/SM/Chol/SM/GM1 (0.4:0.1:0.25:0.25:0.03). Vesicles were approximately 114 nm in diameter for the experiments at both temperatures. Experimental conditions are listed in Table 1 and a Tris buffer system was used. (**A**,**B**) are for experiments at 23 °C, while (**C**,**D**) are for experiments at 35 °C. (**A**,**C**) are the QCM-D frequency and dissipation changes as a function of time, for the different overtones. The circles represent frequency changes, and the triangles represent dissipation. (**B**,**D**) are the ΔD-ΔF plots showing the process of vesicle adsorption taking place and negligible vesicle rupturing at 23 °C and a small fraction of vesicles rupturing at 35 °C.

SLB formation has been generally observed at temperatures above the transition temperature (T_m_) of the lipid species and a recommended strategy to form an SLB is to perform the experiment at approximately 15 °C above the highest T_m_ of the lipid components [49]. In the present study, raising the temperature up to 60 °C did not lead to the complete rupture of vesicles indicating the temperature is not the dominating parameter in vesicle rupture. This is similar to a previous study on adsorption kinetics of DPPC vesicles on SiO_2_ [50] where measurements at a temperature above the DPPC transition temperature did not always lead to vesicle rupture. DPPC vesicles of 90 nm diameter formed a complete SLB at the temperatures about T_m_, but larger vesicles with a diameter of 160 nm did not rupture at the same condition. It was evident that parameters such as vesicle size and vesicle surface coverage on the crystal are also important factors in governing the formation of an SLB [50]. The QCM-D data obtained at 23 °C and 35 °C are compared in Figure 8.

The QCM-D measurements reported in Figure 8A,C show that the peak values of ΔF and ΔD decreased by increasing the temperature, which means there was less vesicle coverage on the quartz crystal surface. This is similar to the behavior observed previously in a QCM-D study of DPPC liposome on SiO_2_ surface [50], where an increase in temperature was shown to lead to stronger vesicle–vesicle interactions and as a result, a lower critical coverage of adhered vesicles is required for vesicle fusion. The QCM-D measurements in Figure 8 also show that increasing the temperature decreased the time of the critical coverage from about 12 min (Figure 8A) to 5 min (Figure 8C). The adsorption of vesicles until the critical coverage is reached is influenced by the diffusivity of the vesicles, and according to the Stokes–Einstein equation, D = kT/(6πηR), (k is the Boltzmann constant, T is the temperature, η is the viscosity of the bulk solution, and R is the vesicle radius), and the diffusivity increases in direct proportion to an increase in the temperature.

The ΔD-ΔF plots in Figure 8B,D show the adsorption kinetic for vesicles at both temperatures. The first process, in which the adsorption of intact vesicles is the dominant process, is similar to the first step in the bilayer formation fingerprint (Figure 2B). However, the second step is different from the bilayer formation fingerprint. In Figure 2B, the second step representing vesicle rupture is indicated by a process tending in the south–east direction. In contrast, in Figure 8B,D, the second process tends north at 23 °C and to north–east at 35 °C, both corresponding to an increase in the softness of the layer. This is indicative of no or partial rupture of the vesicles, and the formation of a soft film.

### 4.4. Vesicle Rupture Increased Appreciably for Much Smaller Vesicles but Remained Incomplete

Reducing the size of vesicles can affect the vesicle rupture in two opposite ways. As mentioned before, vesicle rupture can happen as a response to high membrane bending energy. Therefore, for smaller vesicles with higher curvature energy, the vesicle membrane tension increases, and this promotes rupture of adsorbed vesicles to release the bending energy. On the other hand, adsorption of vesicle on a substrate leads to vesicle deformation which is critical in vesicle rupture [41] as it is shown that greater deformation leads to more favorable rupture. It has been previously reported that vesicles with larger sizes deform to greater extent [43] which means reducing the size of the vesicles can lead to less rupture. The earlier results shown in Figure 8 indicated that changing the vesicle size from 81 nm to 108 nm did not significantly change the extent of vesicle rupture. Therefore, even smaller vesicles, 58 nm in dimeter, were considered.

**Figure 9:**
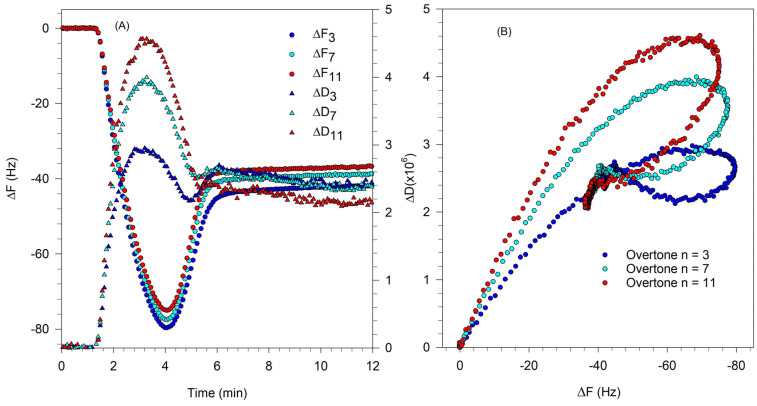
Lipid mixture representing neuronal lipids forming a film of partial bilayer and unruptured vesicles. Experimental conditions are listed in Table 1. Vesicles of size ~58 nm and a phosphate buffer system were used. (**A**) QCM-D frequency and dissipation changes as a function of time for the lipid mixture DOPC/DOPS/SM/Chol/GM1 (0.4:0.1:0.25:0.25:0.03) at 23 °C. The circles represent frequency changes, and the triangles represent dissipation. The different lines correspond to measurements at various overtones. The asymptotic frequency change of about −40 Hz and the high asymptotic dissipation imply that only a part of the adsorbed vesicles ruptured to create a partial bilayer, and the final film was soft and viscoelastic as it included unruptured vesicles. (**B**) The ΔD-ΔF plot shows the process of vesicle adsorption taking place and the partial completion of the vesicle rupture process.

QCM-D measurements shown in Figure 9A indicate that vesicle adsorption takes place leading to a critical saturation value at the quartz crystal surface, indicated by a minimum in the frequency change curve. At this condition, although the rupture of vesicle remained partial, it was much larger than that found in the experiments discussed earlier. The QCM-D signatures of frequency and dissipation changes follow the same trend as that for the formation of an SLB, except that the magnitude of the asymptotic values of ΔF and ΔD are higher than expected for the formation of a complete rigid bilayer. The frequency change ΔF stabilized at a value of about 40 Hz compared to the typical values of about 25 Hz, shown in Figure 2A. The asymptotic dissipation change ΔD was about 2.5 × 10^−6^, indicating that the adsorbed layer is not rigid but soft. The larger asymptotic value of the frequency change coupled to the larger asymptotic value of the dissipation are consistent with a part of the vesicles remaining unruptured and incorporated into the lipid bilayer on the crystal surface. Comparing the ΔF-ΔD plots in Figure 2B and Figure 9B, we see that while the vesicle adsorption process is similar, the vesicle rupture process, although displaying the same qualitative trend as in Figure 2B (a time course towards the southeast), traces only a part of the process.

### 4.5. Osmotic Stress Applied After Vesicle Adsorption Increased Vesicle Rupture, but SLB Formation Remained Incomplete

Since complete vesicle rupture was not realized for the 5-component lipid mixture under the conditions discussed so far, osmotic stress was applied to the vesicles to promote their breakdown. Osmotic stress is caused by having a concentration gradient across the vesicle membrane. The osmotic pressure is denoted positive (hyperosmotic) or negative (hypoosmotic) depending on whether the concentration of salt outside a vesicle is higher or lower, respectively, compared to that inside the vesicle. Positive osmotic pressure causes water flow out of the vesicle and vesicle compression whereby the volume of the vesicle decreases in order to equilibrate the solute concentration inside and outside of a vesicle. As a result of such vesicle deformation induced by hyperosmotic conditions, in addition to the deformation due to vesicle adhesion to the surface, bilayer bending increases, decreasing the barrier for vesicle rupture. By contrast, negative osmotic pressure promotes the expansion of a vesicle because of water entering the vesicle interior, which will counter the volume decrease associated with any vesicle deformation due to surface adhesion. These competing effects may stabilize the vesicle and prevent its rupture. However, a directly contrasting outcome is possible if vesicle swelling can cause pore formation in the bilayer to occur, thereby promoting vesicle rupture.

**Figure 10:**
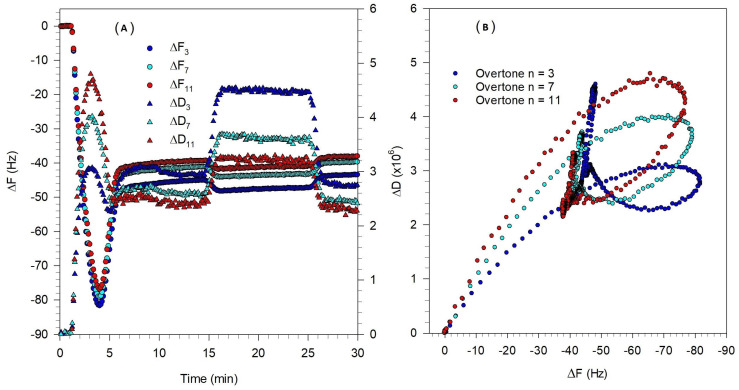
Effect of osmotic pressure applied after vesicle adsorption on the formation of an SLB. Hyperosmotic condition was created with the dilution buffer at a lower salt concentration compared to the rinse buffer. The experimental conditions of buffer compositions and flow times are listed in Table 1. Lipid mixture is DOPC/DOPS/SM/Chol/GM1 (0.4:0.1:0.25:0.25:0.03) at 23 °C. The vesicle diameter is approximately 55 nm. (**A**) QCM-D frequency and dissipation changes as a function of time, for the different overtones. (**B**) ΔD-ΔF plot showing the process of vesicle adsorption taking place and partial bilayer formation along with unruptured vesicles on the quartz crystal surface.

For the experiment designed to apply osmotic stress, we selected vesicles of approximately 55 nm in diameter since the smaller vesicles were found to rupture significantly as discussed in the previous section. The goal was to promote the rupture of those vesicles that remained unruptured. We initially allowed lipid flow under isosmotic condition, by keeping the hydration buffer B2 and the dilution buffer B4 having the same osmolality of 100 mM NaCl. Once vesicles were adsorbed on the surface, the lipid flow was followed by flow of rinse buffer B5 at a higher osmolyte concentration of 250 mM NaCl, to induce hyperosmotic stress on the unruptured vesicle (from 13 to 23 min time points). This was followed by the flow of dilution buffer B4 at 100 mM NaCl (from 23 min to end time points), so that the changes caused by the hyperosmotic stress can be monitored after removing the influence of a change in the buffer on crystal behavior. The QCM-D results are shown in Figure 10.

Figure 10A shows an asymptotic ΔD value of 2.5 × 10^−6^ and ΔF value of −40 Hz, indicating the presence of unruptured vesicles on the crystal surface. The final ΔF and ΔD values at different overtones are spread, indicating a soft layer on the quartz surface incorporating unruptured vesicles and entrapped water along with partially formed bilayer. The results are practically the same as those found in Figure 9 in the absence of osmotic stress. Evidently, this method of inducing osmotic stress, after the vesicles had already adsorbed on the quartz crystal surface and using a rinse buffer with higher osmolality, did not promote the complete rupture of vesicles.

### 4.6. Osmotic Stress Applied During Vesicle Adsorption Causes Total Vesicle Rupture and Complete SLB Formation

**Figure 11:**
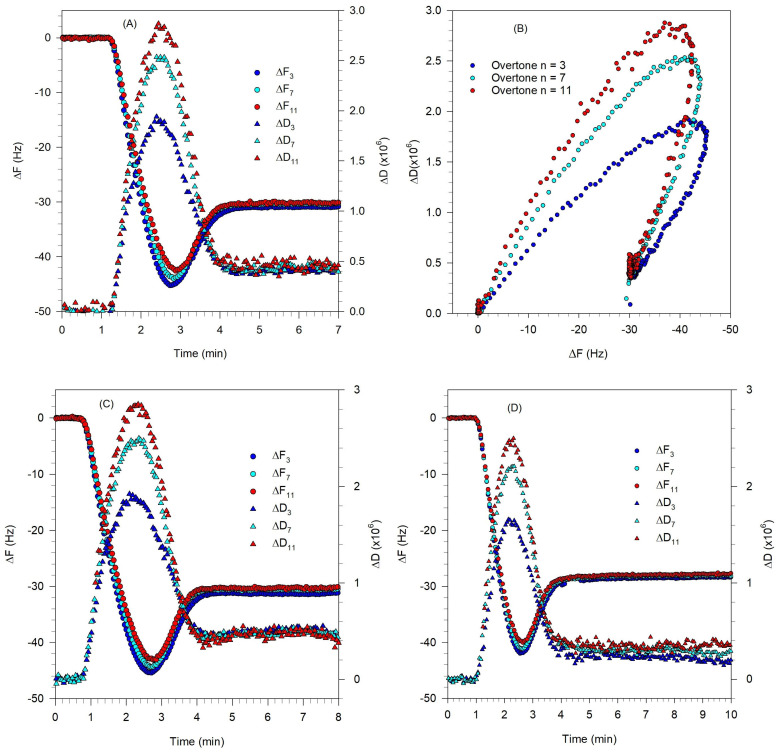
Effect of osmotic stress applied during vesicle adsorption on the formation of an SLB. The osmotic stress was created by using different salt concentrations for the hydration buffer (controlling the salt concentration inside the vesicle) and the dilution buffer (controlling the salt concentration outside the vesicle). The lipid mixture is DOPC/DOPS/SM/Chol/GM1 (0.4:0.1:0.25:0.25:0.03) at 23 °C. The experimental conditions of buffer compositions and flow times are listed in Table 1. (**A**), (**C**), and (**D**) are the QCM-D frequency and dissipation changes as a function of time, for the different overtones. The vesicle sizes are 65, 81 and 74 nm for (**A**), (**C**), and (**D**), respectively. The asymptotic frequency change of about −30 Hz and the small asymptotic value for the dissipation imply the formation of a rigid SLB in all three cases. (**B**) ΔD-ΔF plot corresponding to (**A**) showing the process of vesicle adsorption taking place followed by vesicle rupture process forming a rigid SLB on the surface. The ΔD-ΔF plots for (**C**,**D**) are practically identical to (**B**).

An alternate approach to applying osmotic stress is to introduce the stress during vesicle adsorption. To accomplish this, vesicles made in the hydration buffer were diluted with the dilution buffer that had a higher salt concentration compared to the hydration buffer. The osmolality difference was thus introduced during lipid flow, as the vesicles were adsorbing on the crystal surface. The vesicles were diluted in the dilution buffer just before the QCM-D lipid flow to ensure that the osmotic stress exposure time remains consistent for all experiments. Figure 11 shows such experiments that successfully led to the formation of complete SLBs.

For the experiment described by Figure 11A, the vesicles of approximately 65 nm in diameter were prepared in the hydration buffer B2 (20 mM phosphate, 100 mM NaCl, pH of 7) with the total lipid concentration of 0.3 mM and then diluted to 0.1 mM using the dilution buffer B5 (20 mM phosphate, 250 mM NaCl, pH of 7) with higher osmolality. This approach proved promising as it led to the formation of a complete SLB in every replicate (tested for more than 20 times). As the QCM-D plot indicates, the final ΔF and ΔD values were −30.0 ± 0.1 Hz and 0.50 ± 0.01 × 10^−6^, respectively, slightly higher than those obtained for the DOPC SLB (Figure 2A). These values are comparable with previously reported values from Melby et al. who found ΔF values in the range −25.9 to −29.2 Hz and ΔD values in the range 0.20 × 10^−6^ to 0.52 × 10^−6^ for the SLBs formed from the 3-component lipid mixture DOPC/Chol/SM, as the proportion of cholesterol and sphingomyelin were increased. Figure 11A also shows that the asymptotic ΔF and ΔD responses for different overtones converged, indicating the formation of rigid bilayer. Therefore, the Sauerbrey relation (Equation (1)) can be applied to calculate the mass and thickness of the SLBs.

Taking the Sauerbrey constant C = 17.8 ng/cm^2^/Hz, we calculated the mass of the film on the quartz crystal to be 534 ng. This includes the mass of lipid bilayer as well as the hydration layer between the substrate and the bilayer. The mass and thickness of the hydration layer have been previously reported to be 102 ng/cm^2^ and ~1 nm, respectively [51]. By assuming the density of film to be 1 g/cm^3^, we calculate the thickness of the bilayer as 4.3 nm. This estimate is close to the previous report of 4.1 nm thick SLB for DOPC/DOPS (4:1) from AFM measurements [8] and 4.5 nm thick SLB from egg PC, determined from QCM-D measurements [52]. For the lipid mixture DOPC/DOPS/Chol/SM (1:1:2:2) forming an SLB on mica, An et al. used AFM to determine the thickness of the lipid raft domain to be ~4.8 nm and that of the liquid-disordered domain to be ~5.4 nm [53].

Figure 11C,D describe experiments that followed the same approach of introducing osmotic stress during vesicle adsorption, but using buffers of different osmolalities compared to that for Figure 11A. For the experiments described in Figure 11C,D, vesicles of size ~81 nm (for Figure 11C) and ~74 nm (for Figure 11D) were prepared in the hydration buffer B6 (20 mM phosphate, pH of 7) with the total lipid concentration of 0.3 mM and then diluted to 0.1 mM lipid using the dilution buffer B2 (20 mM phosphate, 100 mM NaCl, pH) which had a higher osmolality. B2 was also used as the rinse buffer. In both experiments, with an osmolality difference of 100 mM NaCl across the vesicle membrane, complete rupture of vesicles and SLB formation occurred similar to the experiment described in Figure 11A where the osmolality difference was 150 mM NaCl. The kinetics of vesicle adsorption and rupturing as indicated by the ΔD-ΔF plot in Figure 11B is very similar to the dynamics observed in Figure 2B for the single component zwitterionic lipid. The ΔD-ΔF plots corresponding to Figure 11C,D are practically similar to the SLB formation dynamics captured in Figure 11B.

### 4.7. Osmotic Stress Applied During Vesicle Adsorption Promotes SLB Formation for 1-, 2-, 3-, 4-, 5-Component Lipid Mixtures

**Figure 12:**
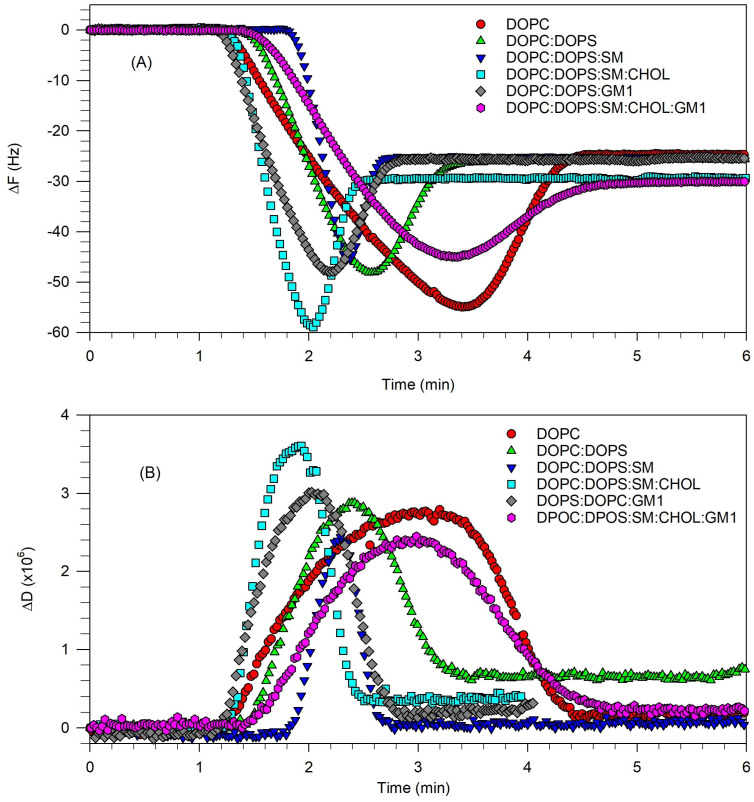
Effect of osmotic stress applied during vesicle adsorption on the formation of an SLB for the following lipid systems: (a) DOPC; (b) DOPC/DOPS (0.40:0.10); (c) DOPC/DOPS/SM (0.40:0.10:0.25); (d) DOPC/DOPS/SM/Chol (0.40:0.10:0.25:0.25); (e) DOPC/DOPS/GM1 (0.40:0.10:0.03); (f) DOPC/DOPS/SM/Chol/SM/GM1 (0.40:0.10:0.25:0.25:0.03) at 23 °C. The osmotic stress was created by different salt concentrations in the hydration buffer (controlling the salt concentration inside the vesicle) compared to that in the dilution buffer (controlling the salt concentration outside the vesicle). (**A**) Frequency change vs. time. (**B**) Dissipation vs time. The plotted data correspond to the 7th overtone QCM-D measurements. The asymptotic frequency change is about −25 Hz for the lipid systems (a), (b), (c), and (e) and about −30 Hz for the lipid systems (d) and (f). In all cases, the asymptotic value for the dissipation is small implying the formation of a rigid SLB in all lipid systems. For all experiments, vesicle interior (hydration buffer B6) is 20 mM phosphate, vesicle exterior (dilution buffer B2) is 20 mM phosphate + 100 mM NaCl and the rinse buffer (B2) is the same as the dilution buffer.

Having identified an approach that enables the effective rupturing of vesicles and formation of a rigid SLB for the 5-component lipid mixture, we have investigated whether this approach based on applying osmotic stress during vesicle adsorption will be effective under various lipid compositions. For this purpose, the method was applied to lipid systems containing 1-, 2-, 3-, 4-, and 5-component systems using the same hydration buffer (B6), dilution buffer (B2), and rinse buffer (B2). The lipid compositions were selected so as to retain the same relative composition as in the 5-component lipid mixture, DOPC/DOPS/SM/Chol/GM1 (0.40:0.10:0.25:0.25:0.03). Namely, for DOPC/DOPS, the mole ratio was 0.40:0.10, for DOPC/DOPS/SM, the mole ratio was 0.40:0.10:0.25, for DOPC/DOPS/GM1, the mole ratio was 0.40:0.10:0.03, and for DOPC/DOPS/SM/Chol, the mole ratio was 0.40:0.10:0.25:0.25. The change in frequency and dissipation measured at the 7th overtone are plotted in Figure 12A and Figure 12B, respectively.

For DOPC, DOPC/DOPS, DOPC/DOPS/GM1, and DOPC/DOPS/SM, the asymptotic frequency change was ~25 Hz and the asymptotic dissipation change was ~0.2 × 10^−6^ (except for DOPC/DOPS, it was slightly higher at 0.5 × 10^−6^), indicating the formation of a rigid SLB. When cholesterol was incorporated as a component as for DOPC/DOPS/SM/Chol and DOPC/DOPS/SM/Chol/GM1, the asymptotic frequency change was ~30 Hz and the asymptotic dissipation change was ~0.2 × 10^−6^, indicating the formation of a rigid SLB. Therefore, the application of osmotic stress during the vesicle flow and adsorption enabled the generation of a fully formed rigid SLB for the various lipid components/compositions tested, making this a general approach to forming SLBs successfully.

### 4.8. In All Cases When SLB Formation Occurred, the Vesicle Deformation Was the Largest

**Figure 13:**
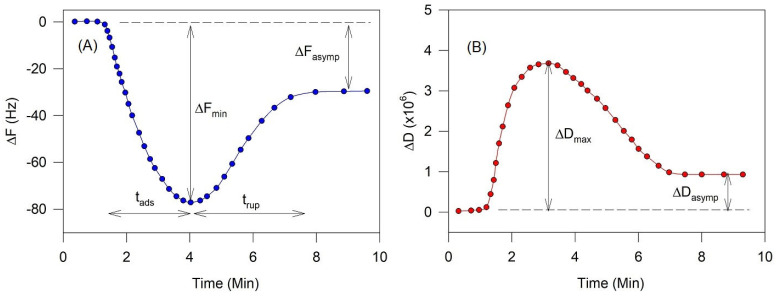
Features extracted for analysis from the QCM-D data of frequency change (**A**) and dissipation (**B**). t_ads_ is the time taken for the adsorption of vesicles, reaching a minimum in the frequency change, denoted as ΔF_min_. The maximum in dissipation also occurs near t_ads_ (but not exactly at t_ads_) and is denoted by ΔD_max_. Following the completion of vesicle adsorption, the time taken for the complete or partial rupture of vesicles is denoted t_rup_ and at this condition the frequency and dissipation changes reach asymptotic values. The asymptotic frequency and dissipation changes corresponding to this condition are denoted as ΔF_asymp_ and ΔD_asymp_.

From the QCM-D responses of frequency and dissipation changes, one can extract the kinetic rates for vesicle adsorption and rupture as well as the thickness of the deformed vesicles on the crystal surface prior to their rupturing. The characteristic QCM-D traces of frequency change and dissipation at one overtone shown in Figure 13 illustrate several critical variables that can be determined from the QCM-D data.

The initial adsorption of vesicles on the quartz crystal surface is denoted by the negative shifts in the resonant frequency (ΔF) of the QCM-D sensor, which correspond to (not directly) mass added to the surface. Typically, a critical density of vesicles must be adsorbed to the surface before the vesicle rupture begins. This corresponds to the minimum in the frequency change curve, ΔF_min_. The time duration over which this minimum is reached from the time the vesicle adsorption starts is denoted as the time of adsorption, t_ads_. During this vesicle adsorption process, the dissipation (ΔD) also increases due to the viscoelasticity of the layer of intact adsorbed vesicles. A maximum in the dissipation, ΔD_max_ is reached near the condition (but not exactly at) where the frequency change shows a minimum. Once the vesicle rupture begins, the magnitude of the frequency change decreases as water is released from the vesicles and eventually an asymptotic value of the frequency change, ΔF_asymp_, is reached. At this condition, either a complete SLB or a partial bilayer with still intact vesicles present among the bilayer patches is realized. Correspondingly, the dissipation begins to decrease from its maximum value and reaches an asymptotic value, denoted as ΔD_asymp_. When a complete SLB is formed, this asymptotic value is closer to zero and when only a partial bilayer is formed with entrapped vesicles among bilayer patches, the asymptotic dissipation change remains large. The time duration from the beginning of vesicle rupture to reaching the asymptotic frequency state is denoted as the time for vesicle rupture, t_rup_.

Values of t_ads_, ΔF_min_, ΔD_max_, t_rup_, ΔF_asymp_, and ΔD_asymp_ extracted from the QCM-D traces for all experiments reported in this paper are summarized in Table 2. All the ΔF values reported are accurate to ±0.2 Hz and all ΔD values are accurate to ± 0.1 × 10^−6^. One can calculate the apparent rate of vesicle adsorption (R_ads_) and the apparent rate of vesicle rupture (R_rup_) using the expressions,

(5) $$R_{ads}=-\frac{{∆F}_{min}}{t_{ads}},\;R_{rup}=-\frac{\left({∆F}_{min}-{∆F}_{asymp}\right)}{t_{rup}}$$

The thickness (h) of the deformed vesicles on the surface is calculated using Equation (4) following the method described in Section 2.3. The frequency change appearing in Equation (4) corresponds to the value of ΔF to which all overtones converge when ΔD/ΔF describing vesicle adsorption are extrapolated to the condition ΔD is zero. The calculated results for R_ads_, R_rup_, and h are also included in Table 2.

**Figure 14:**
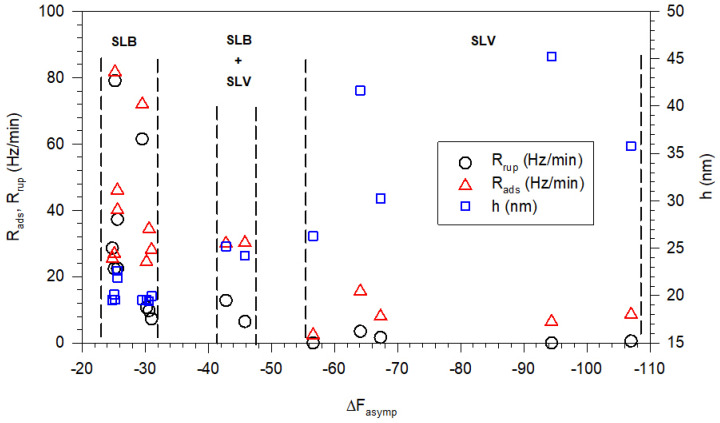
Estimated rates of vesicle adsorption and vesicle rupture and the height of the deformed vesicles in the adsorbed state, all calculated from the QCM-D frequency and dissipation measurements at various overtones, for all the experiments summarized in Table 2. The dotted lines provide the boundaries within which the different structures, SLB, SLB+SLV, and SLV are formed.

The apparent rate of vesicle adsorption (R_ads_), the apparent rate of vesicle rupture (R_rup_), and the thickness (h) of the deformed vesicles on the surface are all plotted in Figure 14 against the corresponding asymptotic value of the frequency change, ΔF_asymp_. The observed frequency changes of ~26 and ~30 Hz, combined with dissipation changes below 0.5 × 10^−6^, correspond to the formation of a complete rigid SLB. The observed frequency changes of 42 to 46 Hz combined with dissipation changes above 1.0 × 10^−6^ correspond to the formation of a soft viscoelastic layer of partial SLB with embedded intact vesicles and entrapped water, on the crystal surface. The observed frequency changes of 55 Hz and above combined with dissipation changes in the range of 1.0 × 10^−6^–20.0 × 10^−6^ correspond to the formation of a supported layer of vesicles (SLVs) with entrapped water in between. In this case, the film on the crystal surface is also soft and viscoelastic. The rate of vesicle adsorption is generally greater than the rate of vesicle rupture. In all cases when an SLB is formed, both the rates of vesicle adsorption and vesicle rupture are larger compared to the case where a complete SLB is not formed. Indeed, when only a supported lipid vesicle (SLV) layer is formed, the rate of vesicle adsorption is the smallest and the rate of vesicle rupture is zero. In all cases when an SLB is formed, the vesicles deform to a thickness of about 20 nm or below.

## 5. Conclusions

Supported lipid bilayers (SLBs) formed on the QCM-D platform have become important model systems to study the biophysical properties of lipid membranes as well as the interactions of various biomolecules with the membranes. In this study, we explored ways of forming an SLB from a multicomponent lipid system representative of neuronal membranes. Neuronal membrane models are important for studies of protein aggregation and fibril formation associated with many neurodegenerative diseases including Parkinson’s and Alzheimer’s disease. QCM-D has become a workhorse to monitor the formation of the SLB as well as to monitor various processes occurring on the membrane including lipid interactions with other molecules and nanoparticles. The robust formation of SLBs on the QCM-D platform has been demonstrated mostly for systems with one or two lipid components. These SLB formation methods are found not to translate well to the multicomponent lipid mixture selected for the neuronal membrane model.

In this study, we explored the conditions for the formation of an SLB, for the five-component lipid mixture composed of zwitterionic DOPC, anionic DOPS, nonionic cholesterol (Chol), zwitterionic sphingomyelin (SM), and anionic ganglioside (GM) by varying experimental parameters such as pH, buffer type, temperature, vesicle size, and osmotic pressure. For most of the variables tested, other than osmotic stress, no or only partial vesicle rupture occurred resulting in the formation of a supported layer of vesicles or an incomplete SLB that included unruptured vesicles. When osmotic stress was applied to the vesicles already adsorbed on the surface, vesicle rupture increased, but it remained incomplete. In contrast, when osmotic stress was applied during vesicle adsorption on the surface, complete vesicle rupture and successful formation of a rigid SLB was demonstrated. The robustness of this approach to form SLB by applying osmotic stress during vesicle adsorption was found to be general for a variety of lipid mixtures composed of 1-, 2-, 3-, 4-, and 5-components.

Each variable we explored in this study such as vesicle size, temperature, change in buffer, lipid concentration, and osmotic stress has been successfully tuned to achieve SLB formation in various 1- or 2-component lipid systems in the literature. What we demonstrate in this manuscript is that the application of osmotic stress during the vesicle adsorption stage enabled SLB formation for the 5-component lipid mixture, whereas the other methods did not lead to an SLB. The osmotic stress-based approach also works well for the 1-, 2-, 3-, and 4-component lipid mixtures and therefore can be viewed as a general way to achieve SLB formation by the vesicle fusion process. While we found this to be the only method that led to successful SLB formation from this 5-component lipid mixture (among the approaches we explored) and while this method can be used for various lipid systems, it is not the only method possible for SLB formation from other lipid systems.

We have also determined the rate of vesicle adsorption, rate of vesicle rupture, and the extent of vesicle deformation on the substrate prior to rupture from the QCM-D measurements. The rate of vesicle adsorption is generally greater than the rate of vesicle rupture. In all cases when an SLB is formed, the vesicles deform to a thickness of about 20 nm or below. If the deformed vesicles are above 20 nm in thickness, we find that only partial or no vesicle rupture occurs. In all cases when an SLB is formed, both the rates of vesicle adsorption and vesicle rupture are larger compared to the case where a complete SLB is not formed. Indeed, when only a supported lipid vesicle (SLV) layer is formed, the rate of vesicle adsorption is the smallest and the rate of vesicle rupture is effectively zero. The osmotic stress applied during the vesicle adsorption process contributes to increasing vesicle deformation, adding on to the deformation induced by vesicle–surface adhesion force, and thereby promotes the vesicle rupture process and the formation of a rigid SLB in a robust way, independent of the number and type of lipid components.

## Figures and Tables

**Table 1:** Experimental systems and conditions reported in this study.

Figure Code	Vesicle Size (nm)	Buffers Composition			Lipid Flow Start/End Time (min)	Rinse Buffer Flow Start/End Time (min)	Temp (°C)
		Hydration (Vesicle Interior)	Dilution (Vesicle Exterior)	Rinse			
2A	118	B1	B1	B1	0–6.5 (B1)	6.5–end (B1)	23
6A	80	B2	B2	B2	0–24 (B2)	24–end (B2)	23
7A	81	B3	B3	B3	0–15 (B3)	15–18 (B3)	23
7C	108	B3	B3	B3	0–15 (B3)	15–18 (B3)	23
8A	114	B3	B3	B3	0–20 (B3)	20–end (B3)	23
8C	114	B3	B3	B3	0–40 (B3)	40–end (B3)	35
9A	58	B2	B4	B4	0–8 (B4)	8–13 (B4)	23
10A	55	B2	B4	B5	0–8 (B4)	8–13 (B4); 13–23 (B5); 23–end (B4)	23
11A	65	B2	B5	B5	0–7 (B5)	7–end (B5)	23
11C	81	B6	B2	B2	0–6.5 (B2)	6.5–end (B2)	23
11D	74	B6	B2	B2	0–6 (B2)	6–end (B2)	23
12A	Various	B6	B2	B2	0–6 (B2)	6–end (B2)	23

Buffer Compositions: (B1) 20 mM phosphate + 100 mM NaCl, pH = 7.8; (B2) 20 mM phosphate + 100 mM NaCl, pH = 7.0; (B3) 10 mM Tris + 150 mM NaCl + 2 mM CaCl_2_, pH = 7.40; (B4) 20 mM phosphate + 100 mM NaCl + 2.5 mM MgCl_2_, pH = 7; (B5) 20 mM phosphate + 250 mM NaCl + 2.5 mM MgCl_2_, pH = 7; (B6) 20 mM phosphate, pH = 7.

**Table 2:** Estimated rates of vesicle adsorption and rupture and height of deformed vesicles.

Figure #	Lipid *	t_ads_ (min)	ΔF_min_ (Hz)	ΔD_max_ (×10^6^)	t_rup_ (min)	ΔF_asymp_ (Hz)	ΔD_asymp_ (×10^6^)	R_ads_ (Hz/min)	R_rup_ (Hz/min)	h (nm)
2A	(a)	2.1	−56.5	2.2	1.4	−25.1	0.1	26.9	22.4	20.1
6A	(f)	23	−56.6	2.8	∞	−56.6	2.8	2.4	0	26.3
7A	(f)	9.1	−73.1	4.5	3.5	−67.3	7.8	8.0	1.7	30.3
7C	(f)	14.7	−94.4	17.2	∞	−94.4	17.2	6.4	0	45.2
8A	(f)	12.8	−110.5	16.9	6.0	−107.0	18.3	8.6	0.6	45.0
8C	(f)	4.9	−76.4	9.2	3.53	−64.1	8.9	15.6	3.6	41.7
9A	(f)	2.7	−79.8	3.0	2.88	−42.8	2.4	30.0	12.8	25.2
10A	(f)	2.7	−81.2	3.0	5.5	−45.8	3.0	30.2	6.4	24.2
11A	(f)	1.6	−45.1	1.9	1.95	−31.0	0.5	28.1	7.2	19.9
11C	(f)	1.4	−47.2	2.0	1.7	−30.6	0.5	34.4	9.8	19.4
11D	(f)	1.6	−42.1	1.7	1.5	27.9	0.3	26.6	9.5	20.7
12A	(a)	2.2	−55.1	2.8	1.06	−24.8	0.1	25.4	28.6	19.5
12A	(b)	1.2	−48.1	2.9	1.0	−25.6	0.7	40.1	22.5	21.9
12A	(c)	0.6	−45.8	2.4	0.26	−25.2	0.1	81.7	79.1	19.6
12A	(d)	0.8	−59.0	3.6	0.48	−29.5	0.4	72.0	61.5	19.5
12A	(e)	1.0	−48.0	3.0	0.6	−25.6	0.2	46.1	37.3	22.6
12A	(f)	1.9	−45.2	2.4	1.4	−30.2	0.2	24.4	10.7	19.6

* For Figure 12A, the lipid systems are: (a) DOPC; (b) DOPC/DOPS (0.40:0.10); (c) DOPC/DOPS/SM (0.40:0.10:0.25); (d) DOPC/DOPS/SM/Chol (0.40:0.10:0.25:0.25); (e) DOPC/DOPS/GM1 (0.40:0.10:0.03); (f) DOPC/DOPS/SM/Chol/GM1 (0.40:0.10:0.25:0.25:0.03). For all other figures, the lipid system is the 5-component lipid mixture, DOPC/DOPS/SM/Chol/GM1 (0.40:0.10:0.25:0.25:0.03).

## Data Availability

Data are contained within the article.
